# *Staphylococcus argenteus* and *Staphylococcus schweitzeri* are cytotoxic to human cells *in vitro* due to high expression of alpha-hemolysin Hla

**DOI:** 10.1080/21505594.2019.1620062

**Published:** 2019-05-25

**Authors:** Cecilia Johansson, Hilpi Rautelin, René Kaden

**Affiliations:** Clinical Microbiology, Department of Medical Sciences, Uppsala University, Uppsala, Sweden

**Keywords:** *Staphylococcus argenteus*, *Staphylococcus schweitzeri*, *Staphylococcus aureus*, virulence, toxin, alpha-hemolysin, hla, agr, quorum sensing

## Abstract

*Staphylococcus argenteus* and *Staphylococcus schweitzeri* are newly identified species of the *S. aureus*-related complex. *S. argenteus,* as occurring globally and showing significant prevalence and comparable infection and morbidity rates compared to *S. aureus*, is becoming clinically important. Whole genome sequencing has revealed the presence of several virulence genes but the molecular mechanisms of *S. argenteus* infection and virulence are largely unknown. Here, we studied the effect of a previously characterized clinical *S. argenteus* isolate on human cells *in vitro*. The clinical isolate, together with the *S. argenteus* type strain MSHR1132T and the *S. schweitzeri* type strain FSA084T, had a cytotoxic effect on the cells, which showed necrotic cell death after a few hours of treatment. The protein causing the cytotoxic effect was purified and identified by mass spectrometry as alpha-hemolysin, Hla, which is awell-known pore-forming toxin in *S.aureus*. The cytotoxic effect could be blocked with an antibody against Hla. *S.argenteus* showed 12–15 fold higher expression levels of *hla* at the RNA level and 4–6 fold higher expression levels at the protein level compared to *S.aureus*. The higher expression levels of *hla* were supported by higher RNA levels of the regulatory factors *sarA* and *saeR*. Also, the RNAIII component of the accessory gene regulator (agr) quorum sensing system was 8,000–10,000 fold higher in the *S.argenteus* isolates compared to *S.aureus*. This is the first study on the effect of *S.argenteus* on ahuman cell line and strengthens the idea of significant virulence of *S.argenteus*.

## Introduction

*Staphylococcus argenteus* was first described in 2009 as part of the *Staphylococcus aureus* clonal complex (CC) 75 [1], but was formally named in 2015 and together with *Staphylococcus schweitzeri* forms the *S. aureus*-related complex, including also CC2250, CC1223, CC2854, and CC2198 [2,3]. *S. argenteus* is globally distributed and has been isolated from both humans and animals [2–6], while *S. schweitzeri* has not been associated with human infections, and has only been isolated from non-human primates [2,7]. Depending on the sample collection, the proportion of *S. argenteus* within the *S. aureus* complex varies from 0.16% to 0.35% in some studies [4,8] to 10% in others [6]. The differences in prevalence may be due to methodological issues as screening methods are mostly still based on *S. aureus*, and more *S. argenteus*-optimized diagnostics might lead to a higher number of positive isolates.

*S. argenteus* is mostly connected to skin and soft tissue infections and has only rarely caused invasive disease [6,9,10]. At first, *S. argenteus* was thought to be less virulent than *S. aureus*, due to the lack of the yellow pigment staphyloxantin [11], which confers resistance against oxidative stress and neutrophil killing [12]. Indeed, *S. argenteus* was later shown to be more susceptible to oxidative stress and neutrophil killing *in vitro* than *S. aureus* and had reduced virulence in murine sepsis and skin infection models [2,3,13], although this was not connected to the deficient staphyloxantin expression [9]. However, as compared to *S. aureus*, similar [3,6] or even higher [14] human mortality rates have been reported for *S. argenteus.*

*S. argenteus* and *S. schweitzeri* have been shown to carry several toxin genes, such as staphylococcal enterotoxins *sea, sec,* and *sed*, hemolysins *hla* and *hlb* and Panton-Valentine leucocidin (*pvl)* [3,7,15-18], although some studies have indicated a lower prevalence of these genes compared to *S. aureus* [3,17]. Using *S. aureus*-specific PCR primers, the majority of *S. argenteus* isolates were first shown to be PVL-negative [3]. However, whole genome sequencing (WGS) later revealed both a nucleotide sequence similarity of only approximately 75% between *S. argenteus* and *S. aureus* in the *pvl* gene, and actually much higher prevalence of *pvl* among *S. argenteus* isolates [18]. Alpha-hemolysin (Hla) was the first staphylococcal exotoxin to be identified and belongs to the group of membrane-damaging toxins (reviewed in [19]), with a well-studied function and role in *S. aureus* virulence. Hla is secreted in a water-soluble form and binds to human cells via its target cell receptor ADAM10 resulting in the formation of heptamer pores in cell membranes. Low levels of Hla result in cell permeabilization, which induces apoptosis both intrinsically through the mitochondrial pathway including caspases 9 and 3 [20], but also extrinsically through TNF-α upregulation and caspase 8 activation [21]. High levels of Hla can also trigger necrosis due to a rapid Ca^2+^ influx [22–24]. Hla, and several other secreted toxins, are regulated by a complex network of factors including SarA, SarT, SaeR, and the quorum sensing accessory gene regulator (agr) system, which is active in late logarithmic to stationary phase of growth [25]. For *hla*, the regulatory RNAIII molecule of the agr system specifically plays an important role by activating both *hla* transcription and translation [26,27].

We previously characterized a clinical *S. argenteus* isolate (RK308) and, using WGS, were able to identify several toxin genes in the genome [18]. To further investigate the molecular mechanisms of *S. argenteus* virulence, we here studied the effect of this particular isolate on human cells *in vitro* and could show that, as opposed to *S. aureus, S. argenteus* was cytotoxic due to high levels of the secreted toxin Hla.

## Materials and methods

### Bacterial isolates and cultivation

The *S. argenteus* clinical isolate RK308 has been described previously [18]. The *S. argenteus* type strain MSHR1132T (isolated from a human sample [11]), and the *S. schweitzeri* type strain FSA084T (isolated from a red-tailed monkey in Gabon, Africa [7]) have been described previously [2] and were purchased from DSMZ, Braunschweig, Germany. The *S. warneri* type strain CCUG 7325T, the *S. haemolyticus* type strain CCUG 7323T and the *S. epidermidis* type strain CCUG 18000AT were purchased from CCUG, Gothenburg, Sweden. The *S. aureus* type strain 1800T and the MRSA reference strain CCUG35601 were also included for comparison. Bacteria were resuscitated from frozen stocks at −70°C onto blood agar and subsequently grown overnight in Müller-Hinton broth at 37°C.

### Cell lines and culturing

The HeLa human cervical cancer and the HT29 human colon cancer cell lines were maintained in RPMI 1640 media (Swedish National Veterinary Institute, Uppsala, Sweden) supplemented with 2 mM glutamine (Gibco by life technologies, Carlsbad, California, US), 10% fetal bovine serum (FBS, Gibco by life technologies), 100 U/ml Penicillin and 100 μg/ml Streptomycin (PeSt, Swedish National Veterinary Institute) and grown at 37°C and in 5% CO_2_.

### Cell infection and cytotoxicity assay

For infection experiments, bacteria from overnight cultures were centrifuged at 8000xg for 5 min, diluted in cell culture media and added to cells at a MOI of 100. For the cytotoxicity assays, the bacterial broth from overnight cultures was instead collected and sterile filtrated through a 0.22 μm filter. Filtrated broth (10–100 μl) was added to cells grown in RPMI 1640 media (Swedish National Veterinary Institute) supplemented with 2 mM glutamine (Gibco by life technologies) and 10% fetal bovine serum (FBS, Gibco by life technologies) and incubated at 37°C and in 5% CO_2_. Un-inoculated broth was used as mock. For neutralization, the anti-Hla antibody (8B7, Abcam ab190467, Abcam, Cambridge, UK) was added at a concentration of 4 μg/ml at the same time as the broth. At 6 h, cells were photographed through the microscope lens.

### MTT assay

The tetrazolium dye MTT (Thiazolyl Blue Tetrazolium Bromide; Sigma-Aldrich (Merck), Darmstadt, Germany) was added directly to cell cultures after 6-h incubation to a final concentration of 200 μg/ml. Cells were incubated for an additional 1–2 h until a sufficient amount of dye had precipitated. Precipitates in the cell culture media were dissolved in equal amounts of 0.04 M HCl in isopropanol and measured spectrophotometrically at 570 nm. One well was set up without cells and used as blank in the spectrophotometer. The viability of the cells was calculated as the inverted absorbance value and compared with cells treated with un-inoculated bacterial broth, which was set to 100%.

### SDS-PAGE and western blot

Bacterial broth was concentrated 10x in PBS on Pierce Protein Concentrator columns (3K MWCO, Thermo Fisher Scientific, Waltham, USA) and run on 4–12% SDS-PAGE gels. Gels were either stained with Coomassie Brilliant Blue or blotted to nitrocellulose filters for western blot. The western blot was blocked with 2% milk in TBS+0.05% Tween and detected with a mouse anti-Hla primary antibody (8B7, ab190467; Abcam, Cambridge, UK) diluted 1:1000 and a goat anti-mouse-HRP secondary antibody (A28177; Thermo Fisher Scientific) diluted 1:2000, both in TBS+0.05% Tween. Bands were visualized using the 1-Step Ultra TMB-Blotting Solution (Thermo Fisher Scientific). The blot was scanned and bands quantified in Photoshop using integrated density measurements.

### Mass spectrometry analysis

Selected bands were excised from the Coomassie Brilliant Blue-stained SDS-PAGE for mass spectrometry analyses. The samples were in-gel digested with trypsin according to a standard protocol and analyzed by LC-Orbitrap MS/MS at the MS Facility, SciLifeLab, Uppsala University. Resulting peptide sequences were matched against all translated ORFs in the sequenced RK308 genome to identify the corresponding full-length ORFs, which were then blasted against *Staphylococcus* proteins to identify the proteins and genes.

### Genomics

All included isolates are previously whole genome sequenced. Sequences for isolates included in cytotoxicity assays and RNA expression analyses are available online in the GenBank database (http://www.ncbi.nlm.nih.gov/Genbank/index.html) under the following accession numbers: *S. argenteus* RK308, LSFQ00000000; *S. argenteus* MSHR1132T, FR821777; *S. schweitzeri* FSA084T, CCEL00000000; *S. aureus* 1800T (DSM20231), AMYL00000000 and MRSA CCUG 35601, REGS00000000. For a complete list of all 116 sequenced *S. argenteus* isolates used to confirm the presence of the *hla* gene, please refer to [18]. Sequence alignments and phylogenetic analyses were done in CLC Main Workbench (Qiagen, Hilden, Germany) using standard program settings.

### RNA preparation from bacteria and cDNA synthesis

Bacteria from overnight cultures were first treated with 4 μg/ml lysozyme in TE buffer for 1 h at 37°. Bacterial RNA was thereafter prepared using TRIreagent (Thermo Fisher Scientific) according to the manufacturer’s protocol. The RNA was DNase treated twice using dsDNase (Thermo Fisher Scientific) with phenol/chisam extraction and ethanol precipitation performed in between. The concentration of the RNA was determined using Nanodrop, and the integrity of the RNA was verified on a 1% agarose gel. A total of 100 ng of RNA was reverse transcribed (RT) using Maxima First Strand cDNA Synthesis Kit for qPCR (Thermo Fisher Scientific) according to the manufacturer’s protocol. A control reaction without RT was set up for each sample to rule out residual DNA. The cDNA synthesis reaction was diluted in water and 1/2000 was used for qPCR. At least three independent RNA preparations were used for qPCR.

### Quantitative PCR

Real-time qPCR was performed in the Bio-Rad CFX96 Touch cycler (Bio-Rad, Hercules, USA) using the DyNAmo HS SYBR Green qPCR kit (Thermo Fisher Scientific). Primer sequences for 16S rRNA, *hla* and regulatory genes are available upon request. For expression analyses, expression levels of each gene were calculated as 2^−CT^, normalized to those of 16S rRNA and expressed as fold over that of the *S. aureus* 1800T isolate.

### Statistical evaluation

Student’s t-test (unpaired, two-tailed, n = 3) was used to evaluate statistical differences between samples as described in figure legends where *** refers to p < 0.001; ** to p < 0.01 and * to p < 0.05.

## Results

### *S. argenteus* and *S. schweitzeri* are cytotoxic to human cancer cells

To assess the effect of *S. argenteus* on human cells, an *in vitro* infection model was used in which HeLa cervical cancer and HT29 colon cancer cells were infected with the *S. argenteus* RK308 isolate and the *S. argenteus* type strain MSHR1132T. For comparison, the *S. schweitzeri* type strain FSA084T, the *S. aureus* type strain 1800T, the MRSA reference strain CCUG 35601, the *S. warneri* type strain CCUG 7325T, the *S. haemolyticus* type strain CCUG 7323T and the *S. epidermidis* type strain CCUG 18000AT were also included in the *in vitro* infection analyses.

**Figure 1.**
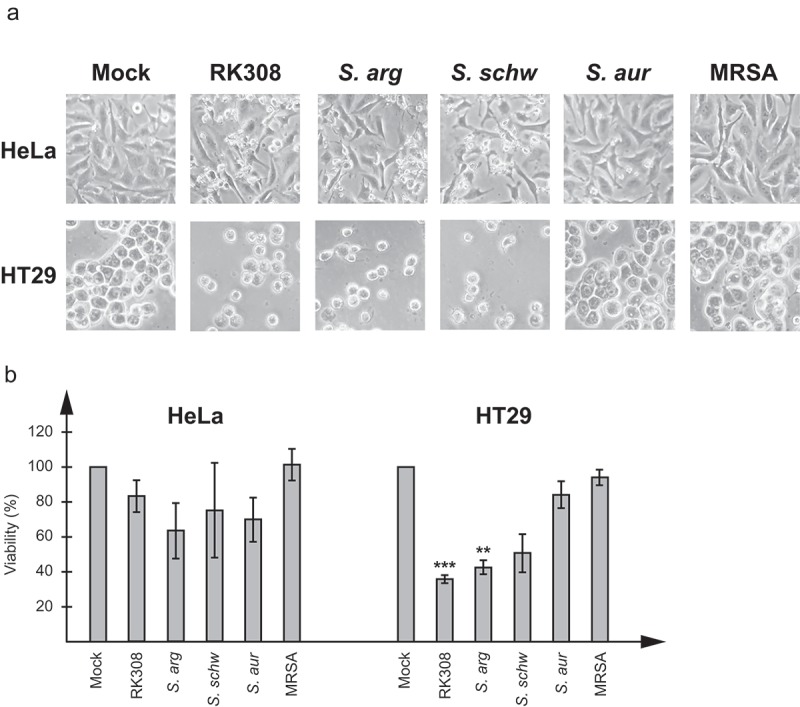
*S. argenteus* and *S. schweitzeri* isolates are cytotoxic to human cancer cells. Broth from overnight cultures of *S. argenteus* RK308 (RK308), *S. argenteus* MSHR1132T (*S. arg), S. schweitzeri* FSA084T (*S. schw), S. aureus* 1800T (*S. aur*) and MRSA CCUG 35601 (MRSA) was added to HeLa and HT29 cells for 6 h. Un-inoculated broth was used as mock. (a) Cells were photographed through the microscope lens. (b) Viability of cells from (a) calculated from the MTT assay compared to mock, which was set to 100%. Asterisks above bars show significant differences as compared to *S. aureus* 1800T.

Both *S. argenteus* isolates and the *S. schweitzeri* isolate had a cytotoxic effect on HeLa and HT29 cells, visible already at approximately four hours after adding bacteria (data not shown). Epithelial cells showed clear signs of cell necrosis with swelling and membrane disruption, which finally led to cell lysis. To see if the effect was caused by a secreted protein, sterile filtered broth from overnight cultures was added to cell cultures. The same cytotoxic effect as seen with living *S. argenteus* and *S. schweitzeri* bacteria was detected (Figure 1(a)). However, none of the other *Staphylococcus* isolates tested showed a cytotoxic effect on the cells, neither with living bacteria nor with broth (Figure 1 and data not shown).

In order to quantify the cytotoxic effect, cell viability was determined by measurement of the cell lysis-induced increase of NAD(P)H-dependent metabolic activity in the cell culture media at 6 h using the MTT assay and compared with cells exposed to un-inoculated broth. For HT29 cells, 40% viability was seen for cells exposed to the broth from *S. argenteus* cultures, while *S. schweitzeri* FSA084T resulted in 50% viability. For HeLa cells, although the cytotoxic effect was clearly visible in the microscope, the measured NAD(P)H-dependent metabolic activity was not as high with neither *S. argenteus* isolates nor with *S. schweitzeri* FSA084T, and resulted in viability of about 60–80%.

### Identification of the cytolysin by mass spectrometry analyses

**Figure 2.**
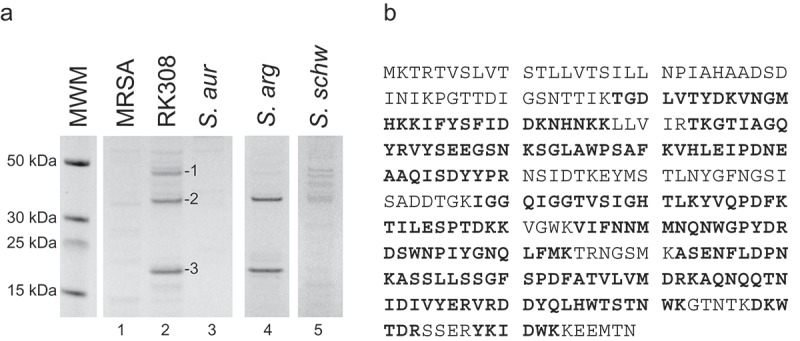
Identification of the cytolysin by MS analyses. (a) Coomassie brilliant blue stained SDS-PAGE of bacterial broth from *S. argenteus* RK308 (RK308), *S. argenteus* MSHR1132T (*S. arg), S. schweitzeri* FSA084T (*S. schw), S. aureus* 1800T (*S. aur*) and MRSA CCUG 35601 (MRSA) used in the cytotoxicity assay. Bands 1, 2 and 3 from the RK308 lane were excised from the gel for MS analyses. MWM, molecular weight marker. (b) Complete amino acid sequence of alpha-hemolysin, Hla, with peptides identified by MS shown in bold.

To identify the cytotoxic protein, broths from overnight cultures used in cytotoxicity experiments were run on SDS-PAGE and stained with Coomassie Brilliant Blue (Figure 2(a)). Three strong bands, of approximately 45, 35, and 20 kDa, respectively, were specific for the RK308 isolate, and two of which were also present in the *S. argenteus* type strain MSHR1132T (Figure 2(a), lanes 2 and 4). These three bands (1, 2, and 3, Figure 2(a), lane 2) were excised from the gel and analyzed by mass spectrometry (MS). The results were matched against all translated ORFs in the RK308 sequence, and each full-length protein was then identified using BLASTp (Table S1).

Band number 1 was identified as a cysteine protease and band number 3 as a DUF4888 domain-containing protein (S1 Table). As none of these proteins were likely to explain the cytotoxic property, they were not further investigated. Instead, band number 2, a protein of approximately 35 kDa and present in both *S. argenteus* and in the *S. schweitzeri* FSA084T isolates, was identified by BLASTp as an *S. argenteus* beta-channel forming cytolysin (Table S1), with 66% coverage in the MS analyses (Figure 2(b) and Table S1). Further BLAST searches on both the gene and protein sequences identified this cytolysin as *Staphylococcus* alpha-hemolysin (Hla).

*In silico* analyses confirmed the presence of the *hla* gene in all 116 *S. argenteus* isolates for which whole genome sequences were available (for a complete list of all sequenced *S. argenteus* isolates included, please refer to [18]). The amino acid sequences of the *hla* genes were almost identical within the *S. argenteus* group. The *hla* gene was also identified in *S. schweitzeri* FSA084T and *S. aureus* 1800T, while not detected in MRSA CCUG 35601, *S. warneri* CCUG 7325T, *S. haemolyticus* CCUG 7323T and *S. epidermidis* CCUG 18000AT used in the experiments.

The *S. argenteus* RK308 Hla protein sequence showed 90% and 89% similarity to *S. schweitzeri* FSA084T and *S. aureus* 1800T, respectively (Fig. S1A). The majority of amino acid residues, earlier shown to be important for binding *S. aureus* Hla to the cell membrane and pore formation [28], were conserved between the two *S. argenteus, S. schweitzeri*, and *S. aureus* isolates (Fig. S1A). However, phylogenetic analyses revealed a closer relationship between *S. schweitzeri* FSA084T and *S. aureus* 1800T while the two *S. argenteus* isolates were more distant (Fig. S1B).

### Neutralization of the cytotoxic effect by an anti-Hla antibody

**Figure 3.**
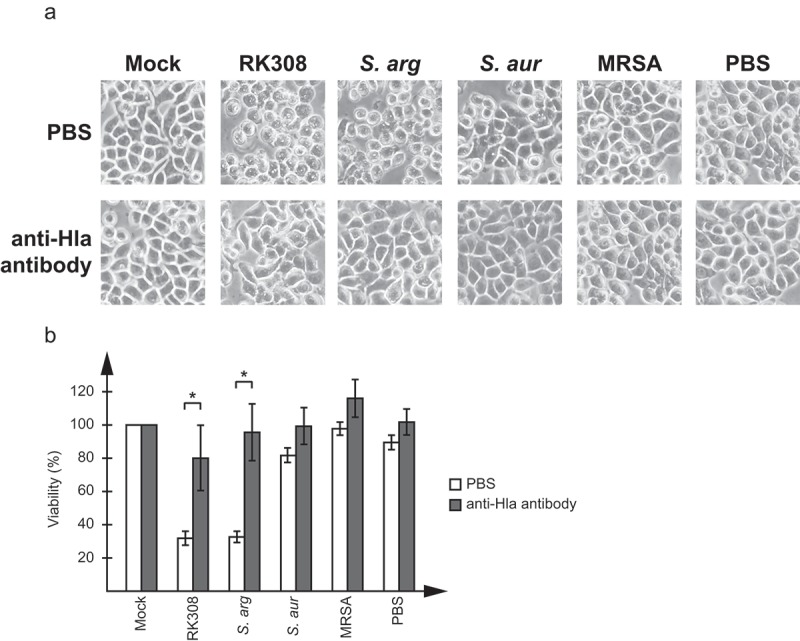
Neutralization of the cytotoxic effect by an anti-Hla antibody. Broth from overnight cultures of *S. argenteus* RK308 (RK308), *S. argenteus* MSHR1132T (*S. arg), S. aureus* 1800T (*S. aur*) and MRSA CCUG 35601 (MRSA) was added to HT29 cells for 6 h together with the anti-Hla antibody or PBS as indicated. Un-inoculated broth was used as mock. (a) Cells were photographed through the microscope lens. (b) Viability of cells from (a) calculated from the MTT assay compared to mock, which was set to 100%. Asterisks show significant differences between antibody- and PBS-treated cells.

To further confirm that the cytotoxic effect was actually caused by Hla, a commercially available antibody against *S. aureus* Hla was tested in neutralization assays on HT29 cells. As shown in Figure 3, the antibody efficiently blocked the cytotoxic effect of the broth added to cell cultures. This could be seen both in the microscope (Figure 3(a)) and as an increase in viability compared to cells only treated with Hla-containing broth (Figure 3(b)).

### *S. argenteus* expresses high levels of *Hla*

**Figure 4.**
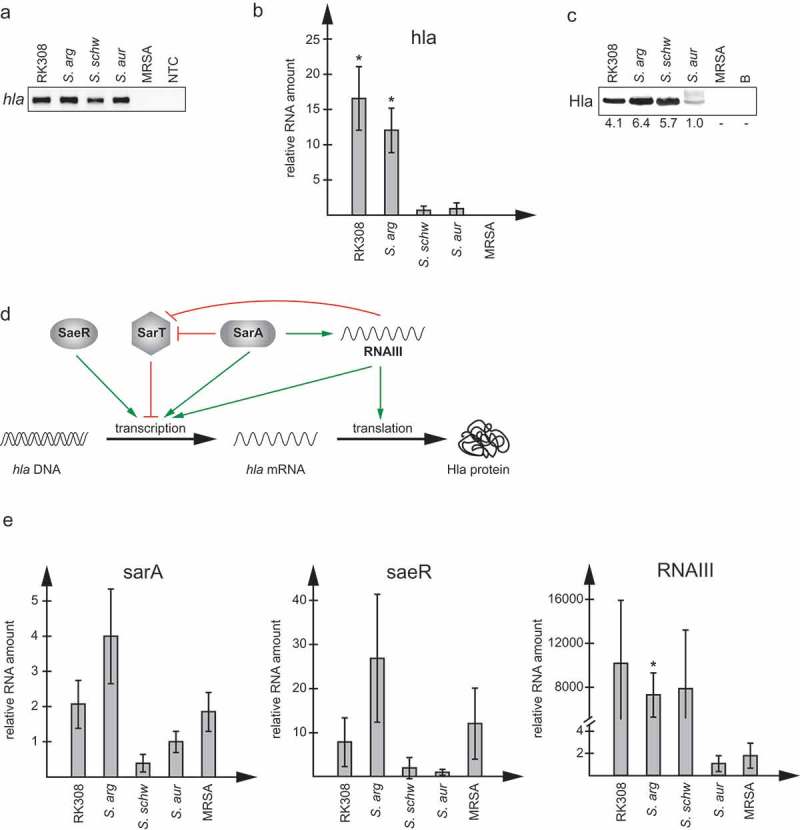
*S. argenteus* expresses high levels of alpha-hemolysin, *hla*. Expression analyses of *hla* and regulatory genes in *S. argenteus* RK308 (RK308), *S. argenteus* MSHR1132T (*S. arg), S. schweitzeri* FSA084T (*S. schw), S. aureus* 1800T (*S. aur*) and MRSA CCUG 35601 (MRSA). (a) Detection of the *hla* gene using PCR. NTC, no template control. (b) RNA levels of *hla* detected with RT-qPCR. RNA expression levels are normalized against 16SrRNA and expressed as fold compared to the *S. aureus* 1800T isolate. Asterisks above bars show significant differences as compared to *S. aureus* 1800T. (c) Hla protein levels in bacterial broth used in the cytotoxicity assay detected by western blot with an anti-Hla antibody. B, un-inoculated broth. Numbers below the blot show fold expression levels compared to *S. aureus* 1800T as quantified in Photoshop. (d) Schematic diagram showing the regulation of *hla* transcription and translation by regulatory factors. Adapted from [25]. (E) RNA levels of regulatory genes *sarA, saeR,* and RNAIII detected with RT-qPCR. RNA expression levels are normalized against 16SrRNA and expressed as fold compared to the *S. aureus* 1800T isolate. Asterisks above bars show significant differences as compared to *S. aureus* 1800T.

The presence of the *hla* gene was confirmed using PCR on genomic DNA. Figure 4(a) shows that *hla* was detected in both *S. argenteus* isolates, as well as in *S. schweitzeri* FSA084T and *S. aureus* 1800T, while not present in MRSA CCUG 35601, in accordance with the *in silico* analyses. The expression pattern of *hla* was thereafter analyzed at both RNA and protein levels, using RT-qPCR and western blotting, respectively. The *S. argenteus* isolates had 12–15 fold higher *hla* RNA levels compared to *S. schweitzeri* FSA084T and *S. aureus* 1800T (Figure 4(b)), resulting in 4–6 fold higher Hla protein levels (Figure 4(c)). Interestingly, *S. schweitzeri* FSA084T had lower *hla* RNA levels, in the same range as *S. aureus* 1800T, but equally high Hla protein levels as the *S. argenteus* isolates included (Figure. 4(b,c)).

To understand the differences in *hla* RNA and protein levels between the included *Staphylococcus* isolates, we measured the expression levels of *saeR, sarA* and the RNAIII component of the agr quorum sensing system, the gene products of which are involved in regulation of *hla* expression at transcription and translation levels (Figure 4(d)). The transcriptional activators *sarA* and *saeR* showed 2–4 fold and 9–28 fold higher expression in *S. argenteus* isolates compared to *S. aureus* 1800T, respectively. RNAIII, which activates both *hla* transcription and translation, showed a remarkable 8,000–10,000 fold higher expression in *S. argenteus* and *S. schweitzeri* isolates compared to *S. aureus*.

## Discussion

Since the discovery of *S. argenteus* in 2009, a lot of the research has focused on distribution in the population [2–6], prevalence rates [4,6,8], presence of virulence genes [3,7,15-18]and challenges in clinical diagnostics as compared to *S. aureus*, while information on *S. schweizeri* is more scarce. This is the first molecular study on the impact of *S. argenteus* and *S. schweitzeri* on human cells *in vitro* and describes a remarkable cytotoxic effect not seen with any other *Staphylococcus* species tested here. In this study, epithelial cells treated with *S. argenteus* and *S. schweitzeri* isolates showed clear signs of cell necrosis with swelling and finally cell lysis, probably caused by membrane disruption, which was further verified as an increase of NAD(P)H-dependent metabolic activity in the cell culture media, and hence reduced cell viability.

Using protein purification, mass spectrometry, and an antibody neutralization assay, we identified the cause of the cytotoxic effect as the secreted Hla toxin. The Hla toxin, which is a well-studied toxin in *S. aureus*, is known to be important for virulence, as it has been shown to directly damage host tissues and disable the immune system by lysing neutrophils [19]. In our study, the *hla* gene was detected in all sequenced *S. argenteus* isolates and was fairly conserved between *S. argenteus, S. schweitzeri* FSA084T and *S. aureus* 1800T. However, the toxin effect showed remarkable differences. As the majority of amino acid residues important for toxin function were conserved, the sequence variation itself was unlikely to explain the difference in the activity of the protein. We therefore decided to compare *hla* expression levels in our isolates. Measurements of *hla* RNA and protein levels in *S. argenteus* bacteria and broth confirmed high levels of both RNA and protein compared to *S. aureus*. Levels of *hla* RNA in *S. schweitzeri* were in the same range as for *S. aureus* while the secreted protein level was equally high as for *S. argenteus*. The high Hla protein levels in *S. argenteus* and *S. schweitzeri* are consistent with the observation that the cytotoxic effect seen was necrosis and not apoptosis, as Hla is known to cause apoptosis at low levels and necrosis at high levels [20–24]. However, it is possible that the cells treated with *S. aureus* broth, which did contain low levels of Hla according to the western blot, had started to show initial signs of apoptosis, although not yet visible in the microscope, or would eventually have developed apoptosis if treatment times had been extended.

The high protein levels of Hla in *S. argenteus* and *S. schweitzeri* were supported by high expression levels of the regulatory factors *sarA, saeR* and *RNAIII* of the agr quorum sensing system. These factors have been shown to have direct effects on both transcription and translation of *hla* in *S. aureus* [25]. The *saeR* factor, which has a direct positive effect on *hla* transcription, was expressed to a 10–30 fold higher level in the *S. argenteus* isolates than in *S. schweitzeri* FSA084T and *S. aureus* 1800T, while *sarA* was expressed at 2–4 fold higher levels, respectively. However, SarA might be expected to have a larger impact on *hla* RNA and protein levels as it both activates *hla* transcription directly and also has an indirect effect on *hla* translation through stabilization of RNAIII. Moreover, SarA also seems to be able to regulate Hla protein levels by regulation of extracellular proteases as *sarA* mutants have been shown to express increased levels of extracellular proteases resulting in lower levels of Hla due to protease-mediated degradation [29]. However, the most striking difference in expression in this study was the 8,000–10,000 fold higher expression level of RNAIII in *S. argenteus* and *S. schweitzeri* compared to *S. aureus*. The agr system is known to be highly active in late-log to stationary phase of bacterial growth, which corresponds to the phase in which the bacterial broth was collected and used in the cytotoxicity assays in this study. There was no difference in *sarA* and *saeR* expression between *S. schweitzeri* and *S. aureus* but, *S. schweitzeri* had the same equally high expression levels of RNAIII as *S. argenteus*, which is likely the reason why *S. schweitzeri* also had such high Hla protein levels.

Hla has for a long time been the target of therapeutic approaches to limit the damage of *S. aureus* infection. This includes strategies such as specific antibodies or small chemical compounds to target not only Hla directly but also regulatory factors of the *agr, sae*, and *sarA* systems and the receptor ADAM10 [30], with the hope that this type of anti-virulence therapies might serve as a complement to antimicrobials. Given the high expression levels of Hla in *S. argenteus* and that many *S. argenteus* isolates are shown to be methicillin-resistant [8,18,31]these approaches might also be important to combat *S. argenteus* infections.

Of particular interest was the extremely high expression levels of RNAIII in the *S. argenteus* and *S. schweitzeri* isolates in this study. RNAIII is expressed from its own promoter (P3) in the agr locus, and further studies are needed to verify whether the other gene products of agr (*agrA-D*), expressed from a different promoter (P2) within the locus, are also increased in *S. argenteus* and *S. schweitzeri*, and whether this contributes to the general characteristics and virulence of these species.

To summarize, we present the first study looking at the effect of *S. argenteus* and *S. schweitzeri* on a human cell line. The findings showing high expression levels of Hla in *S. argenteus* and *S. schweitzeri* and resulting in cytolethal properties contributes to the understanding of *S. argenteus* virulence and support the earlier perceptions of *S. argenteus* being virulent and clinically relevant.

## Data Availability

All sequence data is available in the GenBank database (http://www.ncbi.nlm.nih.gov/Genbank/index.html) under the accession numbers provided above or in(18).
