# Supplementary material for: Staphylococcus argenteus and Staphylococcus schweitzeri are cytotoxic to human cells in vitro due to high expression of alpha-hemolysin Hla
Source: Virulence. 2019 May 25;10(1):502–10. doi: 10.1080/21505594.2019.1620062 (PMC6550535; doi:10.1080/21505594.2019.1620062)

Supplemental information

Table S1. Results from MS analyses

**Gel bands from Fig. 2A**		**Match in RK308 ORF collection**					
#	Estimated size (kDa)	Contig number	number of amino acids	calculated size (kDa)	coverage in MS analysis (%)	number of peptides	Result from BLASTp against *Staphylococcus* proteins
1	45	8	395	45	41	17	Cysteine protease
2	35	8	319	36	66	27	Beta-channel forming cytolysin
3	20	17	189	21	56	11	DUF4888 domain-containing protein (domain of unknown function)

Figure S1.

Hla protein sequence analyses. (A) Alignment of *S. argenteus* RK308 and MSHR1132T, which are identical (*S. arg*), *S. schweitzeri* FSA084T (*S. schw*) and *S. aureus* 1800T (*S. aur*) Hla protein sequences. Dot; identical amino acid to *S. argenteus*, bold; a selection of amino acid residues showed in *S. aureus* to be important for binding to the cell membrane and pore formation, boxed; cleaved signal peptide. (B) Neighbour-joining phylogenetic tree based on alignment of Hla amino acid sequences.

Figure S1.

A

```
                  10        20        30        40        50
                   |         |         |         |         |
S. arg   MKTRTVSLVT STLLVTSILL NPIAHAADSD INIKPGTTDI GSNTTIKTGD
S. schw  ····II·T·· ····LS···M T···N····· ····T····· ·····V····
S. aur   ····I··S·· T···LG···M ··V·N····· ····T····· ·····V····

         LVTYDKVNGM HKKIFYSFID DKNHNKKLLV IRTKGTIAGQ YRVYSEEGSN
         ······I··· ···I······ ·········· ·········· ··········
         ······E··· ···V······ ·······I·· ·········· ··········

         KSGLAWPSAF KVHLEIPDNE AAQISDYYPR NSIDTKEYMS TLNYGFNGSI
         ·········· ··Q······D ·········· ·········· ··T·····N·
         ·········· ··Q·Q·S··· V········· ·········· ··T·····NV

         SADDTGKIGG QIGGTVSIGH TLKYVQPDFK TILESPTDKK VGWKVIFNNM
         TG··S····· L··AS····· ·········· ·········· ··········
         TG··S····· L··AN····· ·········· ·········· ··········

         MNQNWGPYDR DSWNPIYGNQ LFMKTRNGSM KASENFLDPN KASSLLSSGF
         ·········· ·····V···· ·········· ·········· ··········
         V········· ·····V···· ·········· ··AD······ ··········

         SPDFATVLVM DRKAQNQQTN IDIVYERVRD DYQLHWTSTN WKGTNTKDKW
         ·······IT· ····TK···· ··VI······ ····N····· ·····N····
         ·······IT· ····SK···· ··VI······ ·········· ··········

         TDRSSERYKI DWKKEEMTN
         ·········· ·········
         ·········· ·········
```

B

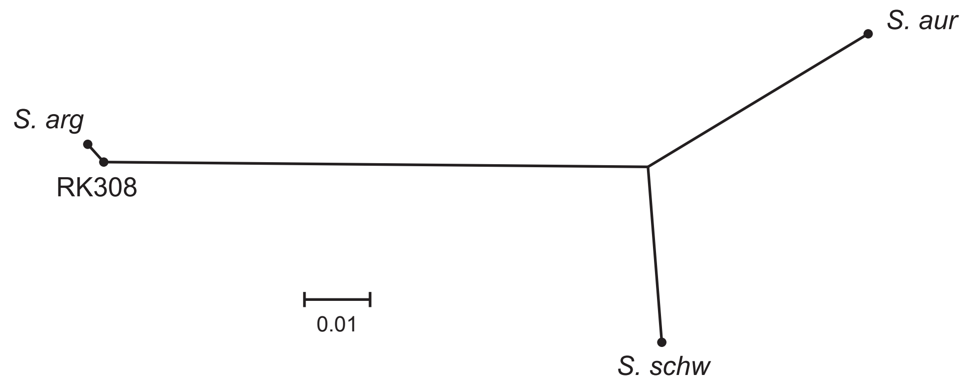

Supplement: Supplemental Material [file kvir-10-01-1620062-s001.pdf]
